# Improving pregnancy outcomes in low- and middle-income countries

**DOI:** 10.1186/s12978-018-0524-5

**Published:** 2018-06-22

**Authors:** Robert L. Goldenberg, Elizabeth M. McClure, Sarah Saleem

**Affiliations:** 1 Department of OBGYN Columbia University, New York, NY USA; 2 RTI International, Durham, NC USA; 3 Aga Khan University, Karachi, Pakistan

**Keywords:** Maternal mortality, Stillbirth, Neonatal mortality, Low-middle income countries

## Abstract

This paper reviews the very large discrepancies in pregnancy outcomes between high, low and middle-income countries and then presents the medical causes of maternal mortality, stillbirth and neonatal mortality in low-and middle-income countries. Next, we explore the medical interventions that were associated with the very rapid and very large declines in maternal, fetal and neonatal mortality rates in the last eight decades in high-income countries. The medical interventions likely to achieve similar declines in pregnancy-related mortality in low-income countries are considered. Finally, the quality of providers and the data to be collected necessary to achieve these reductions are discussed. It is emphasized that single interventions are unlikely to achieve important reductions in pregnancy-related mortality. Instead, improving the overall quality of pregnancy-related care across the health-care system will be necessary. The conditions that cause maternal mortality also cause stillbirths and neonatal deaths. Focusing on all three mortalities together is likely to have a larger impact than focusing on one of the mortalities alone.

## Background

Maternal mortality is generally defined as death to the mother during pregnancy or during the first 42 days after birth. In low and some middle-income countries, maternal mortality rates are 50 to 100-fold higher than those seen in high-income countries [1, 2]. Stillbirth is defined as death in utero after 20 to 28 weeks of pregnancy, depending on where the birth occurs [3, 4]. In low-income countries, stillbirth rates are ten to 20-fold higher than those rates seen in high income-countries. Neonatal mortality is defined as death to a live-born baby within 28 days of birth [5, 6]. The neonatal mortality rates in low and middle-income countries are often tenfold the rates in high-income countries. Many current low-income country mortality rates are similar to the rates in high-income countries from the early 1900’s. This paper first explores the causes of maternal mortality, stillbirth and neonatal death, and then considers why the rates of each outcome have improved in high-income countries. Finally, we review recommendations regarding interventions to reduce these mortalities in low- and middle-income countries.

### Etiology of maternal mortality

In most low and some middle-income countries, the causes of maternal mortality include hemorrhage, hypertensive diseases and maternal infections [7–11]. The timing of hemorrhage is often divided between the antepartum and post-partum periods. The most common cause of antenatal hemorrhage is placental abruption. The most common cause of post-partum hemorrhage is uterine atony. Hypertensive diseases are another cause of maternal mortality. In the first pregnancy compared to subsequent pregnancies, women are at higher risk to develop preeclampsia, a condition marked by hypertension and proteinuria, that may lead to complications including seizures and strokes. If seizures are associated with preeclampsia, the condition is known as eclampsia. Infections contribute to maternal death, but the extent in low and middle-income countries is often not clear. In some classification systems, obstructed labor is not considered a cause of death, with the death generally ascribed to infection or hemorrhage [12]. When women who have an unsafe abortion die, the cause of death is usually defined as either hemorrhage or infection.

### Etiology of stillbirth

For international comparisons of stillbirth rates, the World Health Organization (WHO) recommends using a lower gestational age cutoff of 28 weeks, although in the US and many other high-income countries, lower gestational age cutoffs from 20 to 26 weeks often are used to define stillbirth [3, 13, 14]. In the US, about half of all stillbirths occur at less than 28 weeks [15]; however, this proportion is unknown for low and many middle-income countries. In this paper, we use the terms fetal death and stillbirth interchangeably. In general, we have used the WHO definition that considers stillbirths at 28 weeks gestation or more. In some low and middle-income countries, especially in sub-Saharan Africa, malaria and syphilis are important contributors to stillbirth. However, most fetal deaths are due to asphyxia. Maternal hypertensive diseases, abruption, prolonged labor, breech presentation and umbilical cord accidents are often precursors to asphyxia-related stillbirth [16–21].

### Etiology of neonatal deaths

Neonatal mortality in low and middle-income countries is caused by three major conditions: asphyxia, infection and prematurity [21–23]. Birth asphyxia is usually caused by a maternal condition such as obstructed labor or a placental abruption. Neonatal infections are often associated with maternal infection and are acquired prenatally, although nosocomial infections acquired in the nursery are common. In recent years, it has become clear that prematurity is not the primary cause of neonatal death, but prematurity-related conditions such as respiratory distress syndrome (RDS), a condition caused by failure to produce sufficient quantities of lung surfactant, intraventricular hemorrhage (IVH) or bleeding into the newborn’s brain, and necrotizing enterocolitis (NEC) or breakdown of the infant’s bowel, as well as infection and asphyxia actually cause most of the deaths [22]. Appropriately assigning cause of death for preterm infants is an important step to designing interventions to improve outcomes in preterm infants.

### History of pregnancy outcome improvements in high income countries

There have been large reductions in maternal, fetal and neonatal mortality in high-income and many middle-income countries over the last decades [23–26]. In these countries, until about 1935, maternal mortality ranged from 500 to 1000 deaths per 100,000 live births. Thus, nearly 1% of all mothers died during pregnancy [25]. In recent years, many high-income countries have reported maternal mortality ratios of 10 per 100,000 live births or less [1, 2, 25]. Several papers have explored the history of this rapid reduction that began in about 1935 and was mostly completed by 1970–1980 in nearly all high-income countries. In the 1930s and 1940s, the important interventions introduced included prenatal care and hospitalization for delivery, antibiotics to treat infection, uterotonics, and blood transfusion [26, 27]. Most of the reduction in maternal mortality prior to about 1950 was due to fewer infection-related deaths, probably resulting from the availability and use of antibiotics, although greater attention to infection prevention may have played a role. Although preeclampsia diagnostics including blood pressure testing and testing for proteinuria were made available to many women with the onset of prenatal care, much of the reduction in preeclampsia/eclampsia-related mortality started around 1950. Hospitalization for women with severe or progressive disease became the norm. Not well documented, but probably extremely important, the management of severe preeclampsia and eclampsia transitioned from observation and sedation to rapid delivery [27]. With availability of antibiotics and blood, as well as improvements in anesthesia, cesarean sections became safer and were provided more frequently to terminate pregnancies with prolonged labor or eclampsia and other conditions that were life-threatening to the mother. Thus, beginning in 1935, the introduction of new interventions was responsible for much of the reduction in maternal mortality. With these interventions, introduced first in high-income followed by middle-income countries, nearly 99% of the maternal mortality was eliminated in these regions [26].

Since record keeping began, despite various definitions, in many high-income countries the stillbirth rates approximated 50 per 1000 births [23]. In the 2016 *Lancet* stillbirth series, most high-income countries reported stillbirth rates of < 5 per 1000 births [28]. Several Scandinavian countries and Japan reported rates around 2 per 1000 births. Stillbirth rates began to fall in the 1930’s and continued to decline rapidly until about 1980 [23]. Since 1980, the reductions in most countries has been slower. Because the cause of death for any specific stillbirth is often difficult to determine, it is also nearly impossible to accurately define the interventions responsible for the changes in rates. Nevertheless, some evidence is available. For example, early in the twentieth century, it was estimated that about 20% of US stillbirths were caused by syphilis [29]. Today in most developed countries, syphilis rarely causes a stillbirth but remains a concern in many low-income countries, especially in sub-Saharan Africa [30]. In the early twentieth century, preeclampsia and eclampsia were likely the most important killers of fetuses. However, today those conditions account for only a small percentage of a much smaller number of stillbirths [23]. The risks for stillbirth associated with abruption are much reduced. Monitoring for fetal asphyxia prenatally and during delivery, using various techniques such as fetal heart rate monitoring, with delivery for signs of distress, has reduced much of the asphyxia-related fetal mortality. The high rates of cesarean section and labor induction in many high-income countries occur in part to reduce risk of stillbirth. Thus, with appropriate care for the mother, it has been possible to reduce stillbirth rates substantially [31]. We have also emphasized that the conditions that cause stillbirths and maternal mortality are similar and the interventions that reduce maternal mortality will also reduce stillbirths as well [31, 32].

Neonatal mortality in high-income countries has also decreased since the middle of the last century. There has been a decreasing prevalence of some conditions that cause death but most of the decline is related to better treatment for many of the conditions. Currently in low and middle-income countries, most of the neonatal mortality is divided between asphyxia, infection and preterm birth with a smaller percentage due to congenital anomalies [33]. However, when the infection and asphyxia that occurs in preterm infants is considered, it has been estimated that 60 to 70% of all neonatal deaths occur in preterm infants. In high-income countries, the incidence of preterm birth has remained relatively flat or slightly increased over the last decades, so decreasing rates of preterm birth are not the explanation for the decreased mortality. Preterm infants die from respiratory distress syndrome (RDS), intraventricular hemorrhage (IVH), necrotizing enterocolitis (NEC), infection, as well as asphyxia [22]. Since the middle of the twentieth century, the treatment of RDS with oxygen, and later with various types of ventilation support including continuous positive airway pressure (CPAP), and still later, artificial surfactant, used as either a prevention or treatment, substantially lowered the death rate from RDS. In high-income countries beginning in the 1970’s and continuing through the present, increasing use of maternal corticosteroids prior to delivery substantially lowered the incidence of RDS and the associated IVH, NEC and mortality [34]. The incidence of infection in all neonates was also reduced with better attention to clean delivery care and antibiotics used prophylactically. Treatment with antibiotics for those newborns that did become infected improved. Maternal treatment for syphilis and vaccination for tetanus has reduced newborn infection-related mortality. The incidence of newborn asphyxia was substantially reduced with better obstetric care including monitoring for hypoxia prenatally and during labor, and the increased use of cesarean sections for signs of fetal distress. Newborn resuscitation techniques improved and treatment of newborns with birth-related asphyxia also reduced mortality. Because the care for preterm and sick newborns became increasingly complicated, it was consolidated in newborn intensive care units. Thus, as with maternal and fetal mortality, substantial reductions in neonatal mortality occurred from about 1940 onward.

### Interventions to reduce maternal, fetal and neonatal mortality

The interventions that are effective in preventing most maternal, fetal and neonatal deaths in high-income countries have been well-studied [35]. In theory, if these interventions were available to pregnant women and their newborns in low and middle-income countries, pregnancy related mortality rates should approach those seen in high and some middle-income countries. New interventions or technologies should not generally be necessary to achieve substantial decreases in these mortality rates in low- and middle-income countries. Instead, the question that arises is not what needs to be done, but how to introduce these interventions, ensure their correct use and sustain that use. For example, it has been observed that even if an intervention is available, it may be initiated at the wrong time, poorly implemented or even performed on the wrong patients [34, 36]. Coverage is important, but quality is important as well.

While understanding which interventions are likely to reduce mortality is necessary, ensuring a functioning system of care in which to introduce and sustain a group of interventions is even more important. Single interventions rarely result in substantial reductions in mortality. In order to effectively reduce mortality, it is important to understand the many conditions that cause mortality and the many interventions likely to reduce the mortality as well as defining the population to be cared for, and the resources and personnel available to provide that care.

We next review the interventions that many experts believe should be included in the content of care aimed at reducing maternal, fetal and neonatal mortality in low and middle-income countries. To save the life of a mother, fetus or neonate from a specific condition, the condition must either be prevented or be diagnosed and treated in an appropriate and timely manner. Knowing the conditions mothers, fetuses and newborns die from and when and where they die is therefore crucial. About three quarters of the maternal deaths, about half the stillbirths and about one-quarter of the neonatal deaths occur around delivery [33]. Interventions that improve facility-based delivery and neonatal care will result in the largest reductions in maternal, fetal and neonatal mortality [36].

### Maternal mortality

Especially in south Asia, anemia during pregnancy is a major problem and is in part responsible for many, but an unknown number of deaths. According to data derived from the Global Network’s Registry, in the Pakistani and Indian sites, nearly all women are anemic with a hemoglobin level of less than 11 mg/dl, and in Pakistan, 8 % of the women have a hemoglobin level of less than 8 mg/dl [personal communication]. In a recent publication, anemia has been associated with a doubling of the maternal mortality rate, and low iron levels in the mother and newborn are associated with decreased neurodevelopmental status in the child [37]. Oral iron and folate supplementation have been the mainstays of prevention and treatment strategies to reduce anemia. However, in this supplement, it is argued that since this strategy is often ineffective, other strategies such as intravenous iron treatment are necessary to treat anemia and iron deficiency [38]. It is also important to consider and appropriately treat other causes of maternal anemia such as parasitic infestations.

Hemorrhage, the leading cause of maternal mortality globally, has many different etiologies ranging from an atonic uterus to retained products of conception to lacerations of the cervix, vagina or uterus and uterine rupture. Obstructed labor and unsafe abortions are common precursors of pregnancy-related hemorrhage. A number of maternal medical conditions such as disseminated intravascular coagulation, often linked to infection, can also cause hemorrhage [39]. Cesarean section for prolonged labor can prevent post-partum hemorrhage due to uterine atony. To reduce risk of abortion complications, the best preventative strategy is to have the abortions carried out by skilled practitioners [40]. If hemorrhage occurs, often due to retained products of conception and sometimes a uterine perforation, uterine suction or dilation and curettage (D&C) to evacuate the uterus, blood transfusion, and sometimes abdominal exploration including hysterectomy are necessary to save the woman’s life. Most antepartum hemorrhage is due to either a placental abruption or a placenta previa and in most cases cesarean section is the appropriate treatment for both conditions [39]. Most post-partum hemorrhage is due to an atonic uterus. Use of uterotonics such as misoprostol or oxytocin, and perhaps carbetocin, a log-acting and heat stable oxytocin substitute, given immediately after delivery can prevent many of the post-partum hemorrhages associated with an atonic uterus [41]. However, major reductions in mortality from hemorrhage usually occur with appropriate treatment of hemorrhage once it occurs. Appropriate treatment depends on the cause of the hemorrhage. As above, uterotonics and manual compression of the uterus can reduce ongoing hemorrhage from an atonic uterus. Manual removal of the placenta, uterine suction or D&C can treat bleeding due to retained products, and careful inspection of the vagina and cervix with suturing when necessary can treat hemorrhage from this cause. However, in some cases, perhaps due to a uterine rupture or atonic uterus, failure to respond to conventional treatments can require surgical treatment. Hysterectomy is often the intervention of last resort for many types of hemorrhage unresponsive to other types of treatment. For hemorrhage caused by any of the above conditions, blood products are often also required to prevent mortality. Given the many causes of hemorrhage, we emphasize that accurate diagnosis is needed to identify the appropriate prevention and treatment and therefore, no single intervention is likely to have a large impact on hemorrhage-related maternal mortality.

Infection is the generally considered the second most common cause of maternal death [10]. The most important causes of infection-related maternal mortality are the bacterial infections of the uterus and related sepsis [41]. The reasons for the high risk of infection post-partum are not hard to understand. The vagina is not sterile, and once membranes rupture, vaginal bacteria have easy access to the recently emptied uterus that provides an excellent culture medium for bacteria. Historically, childbed fever often resulted from bacteria transmitted due to lack of handwashing by birth attendants and was the major cause of maternal mortality [42]. In addition to the use of non-sterile delivery techniques, unsafe abortion, prolonged labor, and instrumentation including forceps and cesarean section all increase the risk of infection [40]. Prevention of infection includes use of sterile techniques at delivery and especially provider hand washing, avoiding prolonged labors and reducing instrumentation. Prophylactic antibiotic strategies, especially following cesarean section also prevent post-partum sepsis. Treatment generally consists of the timely administration of appropriate antibiotics but may include D&C to remove infected retained products of conception and in severe cases, hysterectomy. Malaria, also an important infectious cause of maternal mortality in malaria endemic areas, can be responsible for maternal deaths both during acute episodes and increase risk of maternal anemia [43].

In most studies, the third major cause of maternal mortality is hypertension [10, 44]. Most maternal deaths occur following the onset of eclampsia. The causes of maternal preeclampsia/eclampsia are not known. Reduction of risk for preeclampsia/eclampsia has been reported with maternal treatment with calcium and low-dose aspirin, but the percent reductions appear small and almost certainly [45], these interventions will not eliminate this problem. The causes of death in women with preeclampsia and eclampsia include increased placental abruption and disseminated intravascular coagulation, both leading to hemorrhage, asphyxia, aspiration pneumonia, strokes, and cardiac, liver and kidney failure. Preeclampsia is often asymptomatic until seizures or other major complications develop. Therefore, diagnosis through frequent prenatal testing for hypertension and proteinuria is necessary to diagnose the condition, institute effective treatment and reduce mortality. Since preeclampsia/eclampsia is most common late in pregnancy or during the post-partum period, repeated testing during those time periods is crucial if cases are not to be missed. In the end, for progressive disease, the only effective treatment is delivery, but choosing the appropriate time is often problematic. Delivery too soon increases the risk of complications of prematurity in the newborn, while delivery too late increases the risk of stillbirth and the maternal complications described above. If delivery is delayed, close monitoring for worsening disease is required. Delivery is often affected by cesarean section or induction of labor. Antihypertensive therapy may prevent stroke and magnesium sulfate may provide time to allow delivery prior to new or recurrent seizures. Deaths from preeclampsia/eclampsia are nearly all preventable through prenatal care to diagnose the condition, hospital referral for close monitoring, followed by delivery by labor induction or cesarean section. Magnesium sulfate treatment may save some lives but should not be the mainstay of a program to save lives from preeclampsia and eclampsia [26].

In summary, most maternal deaths can be prevented through prevention or early diagnosis of the major conditions that cause the deaths and providing appropriate treatment for those conditions. Since, with a few important exceptions, most complications leading to maternal death cannot be predicted or prevented, care for these conditions must be readily available to all pregnant women. Most maternal deaths occur during labor, delivery and in the immediate post-partum period. When the conditions causing maternal death are considered, it becomes clear that the treatments for these conditions need to be readily available during labor and after delivery. Few of these interventions are available in the home and unfortunately, in most low and middle-income countries, many of these interventions are not available to women delivering in the clinics. For these reasons, having all births occur in hospitals that have these interventions available is the strategy most likely to have the largest impact on maternal mortality.

### Stillbirth

Most stillbirths in low-income countries are attributed to intrapartum events, often associated with poor care at delivery, while intrapartum stillbirths rarely occur in high-income countries [31, 32]. In low-income countries, most stillbirths are due to intrauterine asphyxia, followed by infections and congenital anomalies. Many of the maternal conditions contributing to maternal death also lead to fetal asphyxia. Prolonged/obstructed labor, preeclampsia/eclampsia, placental abruption and pregnancy complications such as growth retardation, abnormal presentations, and cord complications also increase risk for fetal death with the final common pathway being asphyxia. Interestingly, while eclampsia is responsible for most maternal deaths, preeclampsia is most often associated with stillbirth so more intensive monitoring for this condition is advised [26]. Monitoring the fetus for signs of asphyxia, usually by assessing the fetal heart rate either during prenatal care for fetuses at risk or during labor, can determine which fetuses are at risk of stillbirth [45]. Cesarean section for fetuses with heart rate abnormalities or other signs of fetal distress can prevent many stillbirths.

It has been estimated that 25 to 50% of stillbirths are caused by infection [16]. The list of organisms that have been associated with stillbirth is extensive and includes bacteria, viruses, fungi and many parasites. The interventions that may prevent an infection-related stillbirth depend on the organisms involved. Especially in sub-Saran Africa, syphilis is an important cause of stillbirth and programs that diagnose and treat maternal syphilis in that location are important to reduce stillbirth. Malaria causes an unknown number of stillbirths in endemic areas, especially in sub-Saharan Africa, but the impact of malaria in these locations is thought to be large. Preventing maternal malaria with bednets and intermittent prophylaxis are strategies that should reduce malaria-associated stillbirths. Decreasing prolonged labor to reduce bacterial chorioamnionitis, will likely play a role in reducing stillbirths, but the extent of reduction from this strategy is unknown. Similarly, appropriate maternal vaccination for maternal conditions that cause stillbirths such as polio, rubella and other childhood diseases should eliminate some stillbirths. With the possible exceptions of antibiotic use for Group B streptococcus (GBS) or membrane rupture, antibiotics have a smaller role in preventing intrapartum stillbirths.

### Neonatal mortality

Neonates die from three major causes in low and middle-income countries: asphyxia, infection, and conditions related to preterm birth [20]. Asphyxia is best prevented rather than treated. Many of the interventions that decrease maternal mortality and stillbirth also reduce neonatal asphyxia. Since placental insufficiency, cord complications, prolonged labor, abruption and preeclampsia/eclampsia are the maternal conditions most commonly associated with neonatal asphyxia, and the stillbirths generally occur around delivery, fetal monitoring and cesarean section are the strategies generally used to prevent neonatal asphyxia. Mild asphyxia present at birth can be effectively treated by neonatal resuscitation, but newborns with severe birth asphyxia generally require extensive treatment beyond resuscitation and is often of limited success, especially in areas without access to intensive care. We emphasize that prevention of asphyxia should be one of the main strategies used to reduce neonatal mortality.

Approximately one-third of neonatal mortality is directly caused by conditions related to the prematurity itself, while another third of neonatal mortality occurs in preterm infants but is caused by conditions that occur in both term and preterm infants such as asphyxia, infection and congenital anomalies. While there are few if any interventions effective in preventing preterm births, prevention of conditions associated with mortality among preterm newborns has been responsible for much of the remarkable reduction in neonatal mortality over the last decades. In high income-countries, corticosteroids given to the mother in the week prior to delivery may reduce the risk of RDS by 50%, as well as decreasing NEC, IVH and neonatal mortality substantially. As a word of caution, in one low-income study, corticosteroids not only failed to reduce mortality in the targeted infants but was associated with small increases in mortality in term infants and an increase in stillbirths as well [46]. For those neonates with RDS, oxygen, ventilatory support and surfactant substantially reduce mortality and those interventions, especially oxygen and CPAP are thought to have contributed the most to the observed reductions in mortality [34].

Newborn infection is another major cause of mortality. Much of the neonatal mortality from infection is preventable. Vaccination of the mother for conditions such as rubella can prevent neonatal infections resulting in deaths. Deaths from tetanus can be prevented if the mother is immunized. Screening the mother for group B streptococcal infection and treating the mother with antibiotics during labor can reduce much of the neonatal mortality associated with that organism. Attention to hand washing and other techniques to prevent neonatal infection will save many lives. Most important, appropriate use of antibiotics for infants with suspected or confirmed infections has the potential to reduce neonatal mortality substantially.

### General recommendations

International organizations have prioritized interventions appropriate for low and middle-income countries to reduce maternal, fetal, and neonatal mortality. Since most deaths occur around the time of delivery, the United Nations has reviewed this information and promoted several strategies to reduce intra-partum maternal, fetal and neonatal mortality [1]. These strategies include ensuring a skilled birth attendant at delivery, ensuring prompt access to emergency obstetric care and having quality neonatal care including resuscitation available. Ideally, all women would deliver in a facility with essential obstetric and newborn care including intrapartum monitoring with early detection and management or referral to a hospital with advanced capabilities for both maternal and neonatal complications. Since saving many maternal, fetal and newborn lives requires a cesarean section, access to cesarean section in low and middle-income countries is crucial to achieve maternal, fetal and neonatal mortality rates comparable to those seen in high-income countries [47].

The quality of the providers and their level of skills is a crucial consideration. Historically, in the absence of a skilled obstetric provider, maternal mortality approached 1% of all deliveries, rates seen in some low-income countries today. In many low-income countries, until recently, most often the birth attendant was a traditional birth attendant (TBA), traditionally a community woman with little education, but often with practical experience performing deliveries with the practice handed down from generation to generation [48]. In most areas served by TBAs, mortality rates were and remain high. Most studies have shown that even with additional TBA training, the maternal mortality rates do not decline [49]. To reduce those rates close to the levels seen in high-income countries today, a skilled birth attendant is crucial. For this reason, WHO and other organizations have recommended the use of skilled attendants for delivery. However, the training and skills of care-givers designated as skilled attendants vary widely. Many cannot perform a cesarean section, give blood or administer antibiotics. Without these interventions, many women die unnecessarily. We should emphasize that even the most skilled attendant will have little impact on mortality in the absence of blood, uterotonics or antibiotics, facilities and equipment necessary to perform a cesarean section or other life-saving interventions [50].

Another consideration has to do with the quality of data. Historically, in geographic areas where reductions in maternal, fetal and neonatal mortality have occurred, the quality of data has improved as well. In many low and middle-income countries, one of the major obstacles to improving pregnancy outcomes is lack of reliable data on these outcomes. Data that are more difficult to collect, including the causes of death and the coverage for various prevention or treatment interventions are rarely available [51]. Without good quality data, the ability for any treatment facility or geographic area to track their outcomes over time or compare their outcomes with similar entities is problematic. Without good data, evaluating the impact of newly introduced programs or interventions cannot be done. Reliable data are required both to obtain and to document improvement in care and pregnancy outcomes.

A great deal of information exists about the medical interventions that if used correctly would reduce maternal, fetal and neonatal mortality substantially. It is also clear that there is no single intervention that will have a great impact on these outcomes. Instead, a combination of interventions applied at various times during the pregnancy, and especially during labor and delivery, the post-partum period and in the neonatal period, are needed to save lives. Increased access to family planning is of crucial importance. It is also clear that the causes of maternal, fetal and neonatal deaths are often the same and strategies that focus on these conditions will likely have an impact on maternal, fetal and neonatal mortality together [52, 53]. Finally, since many of the conditions that kill mothers, fetuses, and newborns are not easily predicted or prevented, all women and newborns should have rapid access to the many known interventions that save lives. For mothers, fetuses and neonates, these interventions are often hospital-based. Building a system of care by which these interventions can be made available to all women and newborns in a timely fashion must be accomplished if a low or middle-income country is to achieve substantial improvement in its pregnancy outcomes.
